# Stingray-Induced Tarsal Tunnel Syndrome

**DOI:** 10.7759/cureus.16761

**Published:** 2021-07-30

**Authors:** Felicia M Mix, Benjamin M Sucher, Joseph E Brooks

**Affiliations:** 1 Physical Medicine and Rehabilitation, Burrell College of Osteopathic Medicine, Las Cruces, USA; 2 Physical Medicine and Rehabilitation, Electromyography Labs of Arizona Arthritis & Rheumatology Associates, Phoenix, USA; 3 Physical Medicine and Rehabilitation, Genesis Health System, Davenport, USA

**Keywords:** tarsal tunnel syndrome, nerve compression syndrome, stingray injury, electromyography, electrodiagnostic study

## Abstract

This case report involves a 17-year-old male referred for electrodiagnostic (EDX) evaluation with symptoms of tarsal tunnel syndrome after being stung by a stingray. EDX testing revealed moderate axonal injury localized to the tarsal tunnel. Subsequent exploratory surgery removed a stingray barb from the tarsal tunnel. The patient’s symptoms nearly completely resolved at five weeks postoperative follow-up. EDX evaluation of this patient with delayed recovery proved to be a valuable component of management.

## Introduction

Tarsal tunnel syndrome (TTS) is a presentation of symptoms due to an insult to the tibial nerve at the ankle. Multiple causes have been cited such as trauma, compression, systemic, biomechanical, and idiopathic. TTS is clinically diagnosed based on physical examination consisting of burning and tingling in the sole of the patient’s foot, decreased foot sensation, weakness in foot muscle strength, and positive Tinel’s sign over the tarsal tunnel [1]. When paired with physical examination, electrodiagnostic (EDX) studies are a diagnostic modality that localize tibial entrapment mononeuropathy in support of the diagnosis of TTS [2]. This is a case report of a stingray injury inducing TTS, demonstrating how EDX studies play a role in diagnosis and expediting treatment.

## Case presentation

A 17-year-old male presented to Urgent Care with left posterior heel pain where he had been stung by a stingray. The patient received Cefalexin and tetanus vaccination. At a five-month follow-up, the stingray wound had healed, but the patient reported ongoing numbness and an inability to move his lateral two digits. Although he completed a full course of antibiotics, the symptoms in his toes remained. He was clinically diagnosed with sural and deep peroneal neuritis and tenosynovitis of the ankle and foot. In addition to standard of care stingray injury first aid, including soaking his feet with warm water, he received steroid and stem cell injections [3]. Six months after the injury, he was referred for EDX evaluation due to ongoing symptoms.

At the time of EDX evaluation, he reported symptoms of pain, numbness, and weakness presenting in the medial calcaneal region, lateral plantar foot, and lateral two digits. He had difficulty moving his lateral two digits which would intermittently “cramp.” Physical examination revealed a scar in the left medial heel near the Achilles tendon. He demonstrated bilateral full lower extremity range of motion, intact distal pulses, no distal edema, no palpatory restriction or masses around knees and ankles, and intact lower extremity reflexes. The examination was notable for mild left foot weakness (inability to move lateral two digits), mild sensory loss in the left lateral foot, and negative straight leg raise bilaterally. Tinel’s test over the left tarsal tunnel was positive, reproducing radiating symptoms in the lateral plantar and medial calcaneal divisions of the tibial nerve.

A bilateral lower extremity EDX study was performed. Compared to the right, the left lower extremity nerve conduction studies (NCS) revealed low amplitude responses for the left lateral plantar and inferior calcaneal motor branches of the tibial nerve and the left lateral plantar mixed nerve (Table 1). This was consistent with moderate axonal loss injuries at the level of the proximal tarsal tunnel region. No abnormalities were noted in the medial plantar and medial calcaneal nerve branches, and all peroneal and sural nerves tested were within normal limits. Standard proximal tibial motor conduction was normal. Needle electromyographic (EMG) study of the left lower limb was performed with a monopolar electrode and revealed increased membrane irritability and neurogenic firing, consistent with moderate denervation within the left lateral plantar and inferior calcaneal nerve branch distributions. All other muscles examined were within normal limits (Table 2).

**Table 1:** Nerve conduction study findings. mV: millivolts; µv: microvolts; ms: milliseconds

Nerve	Latency		Amplitude	
	L	R	L	R
Lateral plantar (motor)	5.0 ms	3.4 ms	5.1 mV	15.8 mV
Inferior calcaneal (motor)	4.9 ms	3.6 ms	4.8 mV	13.8 mV
Lateral plantar (mixed)	3.3 ms	3.8 ms	4.6 µV	13.4 µV

**Table 2:** Electromyography study findings of the left lower extremity. N: normal

Muscle	Insertional activity	Fibrillations	Positive waves	Firing rate	Recruitment	Interference patterns
Tibialis anterior	N	0	0	N	N	N
Fibularis longus	N	0	0	N	N	N
Tibialis posterior	N	0	0	N	N	N
Extensor hallucis longus	N	0	0	N	N	N
Medial gastrocnemius	N	0	0	N	N	N
Vastus medialis	N	0	0	N	N	N
Fourth dorsal interosseous	Increased	2+	1+	Increased	Decreased	Decreased
Abductor hallucis	N	0	0	N	N	N
Abductor digiti quinti	Increased	2+	0	Increased	Decreased	Decreased

The EDX study revealed left tibial neuropathy of the lateral plantar and inferior calcaneal branches, consistent with TTS. Recommendations were made for podiatry follow-up, advanced imaging with MRI/ultrasound, topical neuropathic pain medication, and surgical exploration for nerve release and possible foreign body removal.

No recommended imaging studies were obtained, but surgery was performed eight months following the initial injury. Surgery included left excision of a foreign body, left tarsal tunnel decompression, left plantar fasciotomy, and treatment of left calcaneal stress fracture. The tibial nerve was noted to have an hourglass appearance with thickened perineurium, consistent with focal compression of the tibial nerve. During dissection of the tarsal tunnel, a foreign body (consistent with a barb from a stingray stinger) was identified and removed. External neurolysis of the tibial nerve and its medial and plantar branches and the first branch of the lateral plantar nerve (the inferior calcaneal nerve) was performed in addition to the decompression of the surrounding fascia. A stress fracture of the calcaneus was observed by the surgeon (unknown etiology, with no imaging documentation), and calcium phosphate was applied. The patient tolerated the procedure well. Postoperatively, the patient was non-weight-bearing and immobilized in a controlled ankle movement (CAM) boot. The patient was advanced to weight-bearing as tolerated in weeks two through five, continuing with the CAM boot. The patient was noncompliant with recommendations to perform physical therapy. At the five-week postoperative follow-up, the patient noted the symptoms to be nearly completed resolved.

## Discussion

TTS has been described as a collection of symptoms due to an insult to the tibial nerve or its branches within the tarsal tunnel. The tibial nerve runs through the tarsal tunnel, which is located from the distal tibia to the plantar aspect of the navicular under the flexor retinaculum (the roof of the tunnel). The symptoms of TTS arise because the tibial nerve provides motor and cutaneous innervation to the foot. The tibial nerve has four terminal branches, namely, the lateral plantar nerve, medial plantar nerve, inferior calcaneal nerve, and medial calcaneal nerve [2]. This anatomic knowledge is critical in forming a differential diagnosis for patients presenting with TTS.

EDX studies are valuable diagnostic tools that evaluate the electrophysiologic function of the tibial nerve and its terminal branches. With a clinical suspicion of TTS, the EDX tests recommended for confirming tibial mononeuropathies include needle EMG and tibial NCS, specifically the medial and lateral plantar motor and mixed nerves, the inferior calcaneal motor nerve, and possibly the medial and lateral plantar sensory nerves [1,2]. In the present case, the needle EMG examination of the lower extremity was useful for ruling out lumbar radiculopathy or lumbosacral plexopathy. EMG examination of specific foot muscles aids in confirming the diagnosis of a tibial versus a tibial branch neuropathy by examining membrane irritability which is consistent with denervation in muscles supplied by the distal branches of the tibial nerve [4]. In the present case, membrane irritability and neurogenic firing were noted in left lateral plantar and inferior calcaneal nerve branch distributions, which can explain the weakness in the lateral foot. The calcaneal fracture noted by the surgeon could have contributed to some weakness secondary to pain, but would not be expected to cause radiating pain with paresthesias and weakness distally into the foot muscles affected by the nerve branches that were injured. This is further supported by the observation of low-amplitude responses on NCS observed for those two nerve branches that innervate muscles controlling the lateral two digits (abductor digiti quinti and fourth dorsal interosseus).

The patient was injured with the barb of a stingray on his left heel. Conservative medical treatment provided minimal symptom relief, making his case suspicious for a retained foreign body causing his neuropathic symptoms. It is important to understand the mechanism of the patient’s TTS injury to understand how EDX studies could play a role in identifying a retained foreign body.

The mechanism of injury from a stingray sting has both a traumatic and toxic component [3]. Injury is due to both punctures of the barb and envenomation. The barb of a stingray is described as a serrated, stiletto-type knife with an integumentary sheath containing venom. The victim of a stingray sting feels immediate intense pain out of proportion to the injury. Edema, discoloration, and necrosis of the wound follow inoculation. Death from a stingray wound is rare and fatalities typically result from the chest or abdomen penetration of the barb into the heart or an abdominal organ. One of the complications of a stingray sting is a retained foreign body. Clinical research shows that hot-water immersion is effective in relieving pain associated with stingray envenomation due to pain pathway inactivation rather than toxin inactivation [3].

In a case of a 29-year-old female with a stingray injury to the ankle causing TTS, MRI was used for confirming the need for surgical exploration [5]. In this patient, MRI revealed tenosynovitis, synovitis, and an effusion, leading the author to speculate the symptoms being due to retained foreign body and prolonged envenomation; and a surgical exploration identified tendon injury and pieces of retained stingray barb [5]. In our case, no preoperative imaging was performed. We believe that the EDX findings of ongoing denervation at six months after injury in a young healthy patient support the hypothesis that prolonged envenomation was an etiologic factor in this patient’s mechanism of injury. Under normal circumstances with acute injury by a foreign body, denervation potentials should subside within three to four months [6]. Ongoing denervation potentials at six months were likely caused by the toxin from the stingray barb (versus the isolated effects of a more benign foreign body on the surrounding soft tissues). This is clinically rationalized based on the ongoing radiation of symptoms distal to the sting site into the foot. Due to the unique injury of a stingray sting, including both trauma from the barb and toxic envenomation, MRI and EDX studies together provide valuable complementary anatomic and electrophysiologic diagnostic information.

## Conclusions

EDX evaluation plays an important role in identifying nerve injury due to foreign bodies causing TTS, such as a stingray barb. Earlier EDX studies can yield an accurate diagnosis. Quicker diagnosis should lead to more effective treatment options by minimizing injury. In this case, surgical exploration confirmed a foreign body contributing to TTS. Repeat EDX study could be useful in comparing pre and postsurgical tibial nerve physiology and healing after the removal of a foreign body. Due to the dual injury mechanism of a stingray injury, pairing MRI with EDX is more valuable than performing one or the other in isolation for the optimum diagnosis of TTS.
